# High-Resolution Magnetic Resonance Black Blood Thrombus Imaging and Serum D-Dimer in the Confirmation of Acute Cortical Vein Thrombosis

**DOI:** 10.3389/fneur.2021.680040

**Published:** 2021-06-21

**Authors:** Si-ying Song, David Dornbos, Duo Lan, Bao-lian Jiao, Shu-ling Wan, Yi-bing Guo, Yu-chuan Ding, Qi Yang, Xun-ming Ji, Ran Meng

**Affiliations:** ^1^Department of Neurology, Xuanwu Hospital, Capital Medical University, Beijing, China; ^2^Advanced Center of Stroke, Beijing Institute for Brain Disorders, Beijing, China; ^3^Department of China-America Institute of Neuroscience, Xuanwu Hospital, Capital Medical University, Beijing, China; ^4^Department of Neurological Surgery, Semmes-Murphey Clinic and University of Tennessee Health Science Center, Memphis, TN, United States; ^5^Department of Neurosurgery, Wayne State University School of Medicine, Detroit, MI, United States; ^6^Department of Radiology, Beijing Chaoyang Hospital, Capital Medical University, Beijing, China

**Keywords:** cerebral cortical venous thrombosis, magnetic resonance black-blood thrombus imaging, D-dimer, high-resolution MRI, diagnosis, batroxobin

## Abstract

Cerebral cortical vein thrombosis (CCVT) is often misdiagnosed because of its non-specific diagnostic symptoms. Here, we analyzed a cohort of patients with CCVT in hopes of improving understandings and treatments of the disease. A total of 23 patients with CCVT (confirmed with high-resolution imaging), who had been diagnosed between 2017 and 2019, were enrolled in this cohort study. Baseline demographics, clinical manifestations, laboratory data, radiological findings, treatment, and outcomes were collected and analyzed. Fourteen females and nine males were enrolled (mean age: 32.7 ± 11.9 years), presenting in the acute (within 7 days, *n* = 9), subacute (8–30 days, *n* = 7), and chronic (over 1 month, *n* = 7) stages. Headaches (65.2%) and seizures (39.1%) were the most common symptoms. Abnormally elevated plasma D-dimers were observed in the majority of acute stage patients (87.5%). The diagnostic accuracy of contrast-enhanced magnetic resonance venography (CE-MRV) and high-resolution magnetic resonance black-blood thrombus imaging (HR-MRBTI) in detecting CCVT were 57.1 and 100.0%, respectively. All patients had good functional outcomes after 6-month of standard anticoagulation (mRS 0–1) treatment. However, four CCVT patients that had cases involving multiple veins showed symptom relief after batroxobin therapy (*p* = 0.030). HR-MRBTI may be a fast and accurate tool for non-invasive CCVT diagnosis. HR-MRBTI combined with D-dimer can also precisely identify the pathological stage of CCVT. Batroxobin may safely accelerate cortical venous recanalization in combination with anticoagulation. Follow-up studies with larger sample sizes are suggested to evaluate the safety and efficacy of batroxobin for treating CCVT.

## Introduction

Cerebral venous system thrombosis commonly involves the cerebral venous sinuses (CVS), deep cerebral veins, and cerebral cortical veins (CCV). Cerebral cortical vein thrombosis (CCVT) shares similar predisposing conditions with cerebral venous sinus thrombosis (CVST), and they are frequently comorbid. However, CCVT is easily misdiagnosed due to its non-specific clinical presentation and confounding radiological findings. These factors can also lead to delays in treatment. Isolated cortical vein thrombosis (ICoVT) is an exceedingly rare condition that represents ~6% of all cerebral venous thrombosis (CVT) entities (1, 2).

Previously limited to case reports and small case series, CCVT diagnoses are mainly based on clinical symptoms and routine imaging features (3). Seizures are one of the most common symptoms of CCVT (1, 4–6). Additionally, complications such as hemorrhagic infarctions (4), subarachnoid hemorrhages (SAH) (7, 8), and arteriovenous fistulas (AVF) (9–11) may arise because of treatment delays.

There are currently no reported guidelines for rapid and precise identification of CCVT, and difficulties confirming CCTV diagnoses persist, for a few reasons. First, patients with CCVT typically present with non-specific symptoms and signs, such as headaches, seizures, and nausea/vomiting. The non-specific nature of this presentation often results in misdiagnosis at the initial onset of the disease process. There is also no clear or conventional imaging modality that allows CCVT to be confirmed rapidly or accurately, which leads to treatment delays. Additionally, even after CCVT has been diagnosed, treatment difficulties persist. Thrombectomy is not a suitable treatment for removing slender cortical vein thrombosis, local thrombolysis may not easily reach effective concentrations because of compensatory varicose cortical vein formation, and the efficacy of anticoagulation monotherapy is uncertain.

Our previous study revealed that abnormally elevated plasma D-dimer counts could predict acute CVST (12). However, the diagnostic value of D-dimers in acute CCVT is still not clear. We have also developed a high-resolution magnetic resonance sequence (high-resolution magnetic resonance black blood thrombus imaging, or HR-MRBTI), which appears to predict CVST (13). Nevertheless, its utility for CCVT diagnosis has not yet been confirmed. With regard to CVST treatment, we previously found that batroxobin, combined with anticoagulation, can safely and effectively improve clinical outcomes and reduce restenosis in patients with acute CVST (14, 15). To evaluate the clinical applications of this novel imaging sequence in diagnosing CCVT, and to evaluate the efficacy of batroxobin for treating CCVT, we conducted a cohort study of Chinese patients with CCVT.

## Methods

### Patients

For this retrospective cohort study, we enrolled 23 patients who had been diagnosed with CCVT and were admitted to the Neurology Department of Xuanwu Hospital, associated with Capital Medical University, in Beijing, China, from 2017 through 2019. The study was approved by the ethics review board (Number: Clinical research 2019-006). Contrast-enhanced magnetic resonance venography (CE-MRV), HR-MRBTI, or digital subtraction angiography (DSA) was used to confirm CCVT. All patients underwent baseline peripheral blood D-dimer testing and routine laboratory and radiological examinations, including cerebral computer tomography (CT) and magnetic resonance imaging (MRI). Outcome measures included clinical examinations, HR-MRBTI, and lumbar punctures to assess intracranial pressure (ICP). Cutoff values for laboratory results were based on referential intervals in the Laboratory of Xuanwu Hospital at Capital Medical University (Supplementary Table 1).

Demographic information, risk factors, presenting symptoms, clinical signs, treatment, and outcomes were analyzed. Laboratory and radiological findings, including CT, MRI, HR-MRBTI, CE-MRV, and DSA were collected from our inpatient database. The association between baseline plasma D-dimer and HR-MRBTI results was evaluated and used to analyze differences in the pathological stages of CCVT.

Modified Rankin Scale (mRS) scores were used to evaluate the functional outcomes of the patients at the time of discharge, and the Patient Global Impression of Change (PGIC) score was assessed to predict outpatient follow-up outcomes. PGIC is a semi-quantitated self-evaluation scale that assesses patients' overall symptom changes using a 7-point scale (1 = very much improved, 2 = much improved, 3 = minimally improved, 4 = no change, 5 = minimally worse, 6 = much worse, 7 = very much worse).

### HR-MRBTI and Imaging Evaluation

All MRI studies were conducted on a 3.0T system (MAGNETOM Verio, Siemens Healthcare, Erlangen, Germany) using a 32-channel head coil for signal reception. Typical imaging parameters of HR-HR-MRBTI included: oblique coronal single-slab coverage, repetition time (TR) = 800 ms, echo time (TE) = 22 ms, matrix = 198 × 192, FOV = 160 × 200 mm^2^, slice thickness = 0.6–1.0 mm, slices = 100–200, scan time = 6–8 min. Detailed information about the HR-MRBTI procedure can be found in our former study (13).

All HR- images were randomized and presented to two independent readers (SY-S and D-L). The readers were not involved with the diagnostic and/or therapeutic management of patients and were blinded to clinical information and conventional imaging data on which the CCVT diagnosis was based.

### Statistical Analysis

Continuous data following a Gaussian distribution are presented as mean ± standard deviation, and categorical data are expressed as *n* (%). *T*-tests or Fisher exact tests were used to compare continuous and/or categorical variables between patients who were and were not treated with batroxobin. Stata software (version 15.0 SE) was used for all analyses. Two-sided *P* < 0.05 were considered statistically significant.

### Data Availability

Anonymized data used here will be shared upon reasonable request to qualified investigators.

## Results

### Clinical Manifestations

This cohort included 14 females and nine males with a mean age of 32.7 ± 11.9 years (range: 17–62 years). Patients presenting within 7 days of symptom onset (acute stage) accounted for 39.1% (9/23) of cases, whereas 30.4% (7/23) of patients presented in the subacute stage (8–30 days) and 30.4% (7/23) presented over 30 days (chronic stage) following the onset of symptoms (Table 1).

**Table 1:** Clinical baseline information of the patients with CCVT on their admissions.

**Case number**	Age^*^, sex	**Clinical symptom**	**Interval^#^**	**Neurological examination**	**Previous medical history**	**Abnormal laboratory investigation**	**Lumber puncture pressure (mmH_**2**_O)**
1	25, F	Generalized Seizure	30 min	Left-side hemiparesis, left side Babinski (+)	Postpartum	WBC↑ Neutro↑ CRP↑ Hs-CRP↑ PS↓ DD↑ Total T3, T4↑ NSE↑	330 +
2	30, F	left-side hemiparesis	8.5 h	Left-side hemiparesis; left side Babinski (+), papilledema (Frisen scale 2)	OCP use, hyperfibrinogenemia	WBC↑ Neutro↑ Hb↓ CRP↑ Hs-CRP↑ IL-6↑ DD↑ Total T3, T4↑	Non
3	43, F	Headache, nausea, dizziness	22 days	Right-side hemiparesis	OCP use, PS deficiency, PC deficiency, abnormal thyroid function	Lym↑ PLT↑ PS↓ PC↓ AT-III↑ TPO-Ab↑ ANA (+)	165
4	24, M	Headache, generalized seizure	30 min	Papilledema (Frisen scale 2)	Alcohol intake history; Recent pneumonitis history	Hs-CRP↑ UC↑ TG↑ LDL↑ PS↓	260
5	45, M	Generalized seizure	1 month	No positive finding (Frisen scale 0)	HBP, smoking, alcohol intake	VitB12↓	150
6	49, F	Generalized seizure	3 months		Migraine, HBP	TG↑ LDL↑ HDL↓	210
7	20, M	Left-side hemiparesis and generalized seizure	6 days	Left-side hemiparesis, bilateral side Babinski (+)	Parotid carcinoma treated with 3-year radiotherapy, hyperthyroidism	WBC↑ Neutro↑ PLT↑ Hs-CRP↑ IL-6↑ DD↑	120
8	62, M	Left-side hemiparesis and focal seizure in left lower limb	20 days	Left-side hemiparesis	HBP, hyperlipidemia, otitis media.	RDW↑ hs-CRP↑ PS↓ PC↓ IgE↑	150
9	17, M	Headache, blurry vision, nausea, left-side hemiparesis and generalized seizure	7 days	Left-side hemiparesis, bilateral papilledema (Frisen scale 4)	PS deficiency, PC deficiency.	PS↓ PC↓ DD↑ Homocysteine↑	330+
10	20, F	Headache	5 days	No positive finding	Post hemorrhoid surgery	DD↑	Non
11	28, F	Headache, blurry vision	4 months	Bilateral papilledema (Frisen scale 3)	Recent mastitis history	PLT↑ Homocysteine↑ Tg-Ab↑	330+
12	38, F	Headache	24 days	Bilateral papilledema (Frisen scale 1)	Non	PLT↑ ESR↑ Tg-Ab↑ TPO-Ab↑	Non
13	27, M	Headache and dizziness	1 year	No positive finding (Frisen scale 0)	Non	Homocysteine↑	195
14	18, F	Headache, dizziness and nausea	4 years	No positive finding (Frisen scale 0)	PS and AT-III deficiency	PS↓ AT-III↓	170
15	35, F	Headache, nausea/vomiting, blurry vision	1 day	Bilateral papilledema (Frisen scale 4)	APS, mild anemia.	RBC↓ Hb↓ Homocysteine↑ DD ↑	300
16	53, F	Headache, blurry vision	1 year	Bilateral papilledema (Frisen scale 1)	Non	Tg-Ab↑ ANA (+)	Non
17	37, M	Headache, dizziness	20 days	Bilateral papilledema (Frisen scale 2)	HBP, hyperlipidemia, smoking, alcohol intake	PC↓ AT-III↓ DD↑	300+
18	31, M	Headache, dizziness and loss of consciousness	4 days	Right eye papilledema (Frisen scale 2)	Nephrotic syndrome, hyperuricemia, hyperlipidemia, hypoalbumia, hypothyroidism	TG↑ LDL↑ HDL↓ Fig, DD↑ BNP↑ IL-6↑ WBC (CSF)↑ Neutro↑	320
19	37, F	Headache, right-side hemiparesis and generalized seizure	15 days	Right-side hemiparesis	Anemia, chronic HBV, subclinical hyperthyroidism	AT-III↓ PLT↑ WBC (CSF)↑ IgG (CSF)↑	125
20	35, F	Headache, nausea/vomiting	8 days	No positive finding	Previous CVST history (1.5 years), APS	DD↑ PLT↑ AMA2↑	Non
21	32, F	Headache	1 month	Bilateral papilledema (Frisen scale 3)	Non	PS↓ AT-III↓	150
22	18, M	Left-side hemiparesis and generalized seizure	1 day	Left-side hemiparesis	Idiopathic thrombocythemia	DD, PLT↑ WBC, Neu↑ Homocysteine↑	Non
23	27, F	Vision blurry	5 days	Bilateral papilledema (Frisen scale 2)	Obesity, HBP, suspected SLE,	DD↑ Hs-CRP, IL-6↑ LA, ESR↑ Neu, RDW, PLT↑	430

*CCVT, cerebral cortical venous thrombosis; F, Female; M, male; WBC, white blood cell; Neutro, Neutrophil; Lym, lymphocyte; PLT, platelet; Hb, hemoglobin; RBC, red blood cell; CRP, C-reactive protein; IL-6, Interleukin-6; Hs-CRP, high-sensitivity C-reactive protein; DD, d-dimer; PS, Protein S; PC, Protein C; AT-III, antithrombin-III; T3, triiodothyronine; T4, Thyroxine; Tg-Ab, thyroglobulin antibodies; NSE, Neuron-specific enolase; TPO-Ab, Thyroid peroxidase antibody; ANA, antinuclear antibody; ESR, erythrocyte sedimentation rate; TG, triglycerides; LDL, Low-density lipoprotein cholesterol; HDL, High-density lipoprotein cholesterol; UC, uric acid; APS, Antiphospholipid syndrome; AMA, anti-mitochondria antibody; HBP, high blood pressure; OCP, Oral contraceptives; Non, none. ↑, higher than upper reference level; ↓, lower than lower reference level*.

* *Age at developing CCVT (years)*.

# *Interval between symptoms occurrence and admission (days)*.

Present risk factors within this cohort included hyperhomocysteinemia (21.7%), hypertension (21.7%), hyperlipidemia (13.0%), history of alcohol abuse (13.0%), abnormal thyroid function (13.0%), and being post-surgery (8.7%). Two out of the 14 female patients used oral contraceptives (14.3%). Many patients presented with headaches (65.2%) and seizures (39.1%). Seizures were large of the generalized tonic colonic type (88.9%). Further clinical manifestations of CCVT included visual disturbance (21.7%) and hemiparesis (30.4%).

### Laboratory Features

Abnormally elevated inflammatory biomarkers were identified in 65.2% (15/23) of our patients. Inflammatory markers included increased white blood cell (WBC) counts (17.4%), abnormally elevated neutrophil counts (17.4%), interleukin-6 (IL-6) (17.4%), C-reactive protein (CRP) (13.0%), and hypersensitive-CRP (hs-CRP) (34.7%). Vasculitis evaluation, including antinuclear antibody (ANA), anti-neutrophil cytoplasmic antibody (ANCA), and antiphospholipid antibody (APLA), was negative in 19 cases. The remaining four cases were suspected to have underlying autoimmune diseases. Two patients were diagnosed with antiphospholipid syndrome, one patient had a positive level of anti-mitochondria antibodies, and the other patient was found to have high ANA titers (1:320) (Table 1).

More than 85% of CCVT cases in the acute stage (8/9) presented with abnormally elevated plasma D-dimers. Hypercoagulation status was identified in several cases, including five (21.7%) with Protein S deficiency, six (26.1%) with Protein C deficiency, five (21.7%) with antithrombin III (AT-III) deficiency, and two (8.9%) with hyperfibrinogenemia. Eight (34.8%) patients were found to have abnormal thyroid functioning, including elevated thyroid peroxidase antibodies (17.4%), increased anti-thyroglobulin antibodies (13.0%), and increased total triiodothyronine and thyroxine (8.7%). Seventeen patients underwent lumbar punctures to assess intracranial pressure (ICP), which was found to be over 250 mmH_2_O in eight (34.7%) cases (Table 1).

### Imaging Presentations

**Figure 1:**
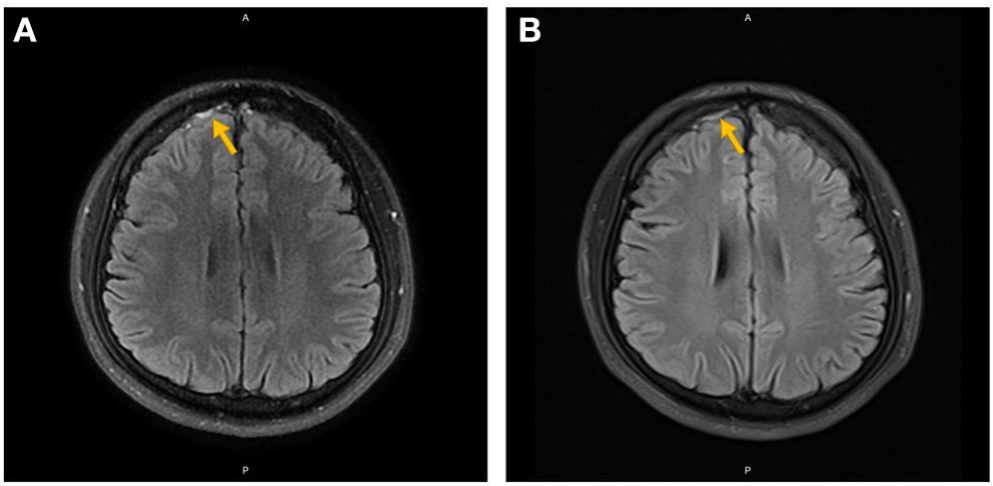
Non-enhanced **(A)** and contrast-enhanced **(B)** MRI of the brain in Case 4. The orange arrow indicates subarachnoid hemorrhage in the right frontal lobe.

**Figure 2:**
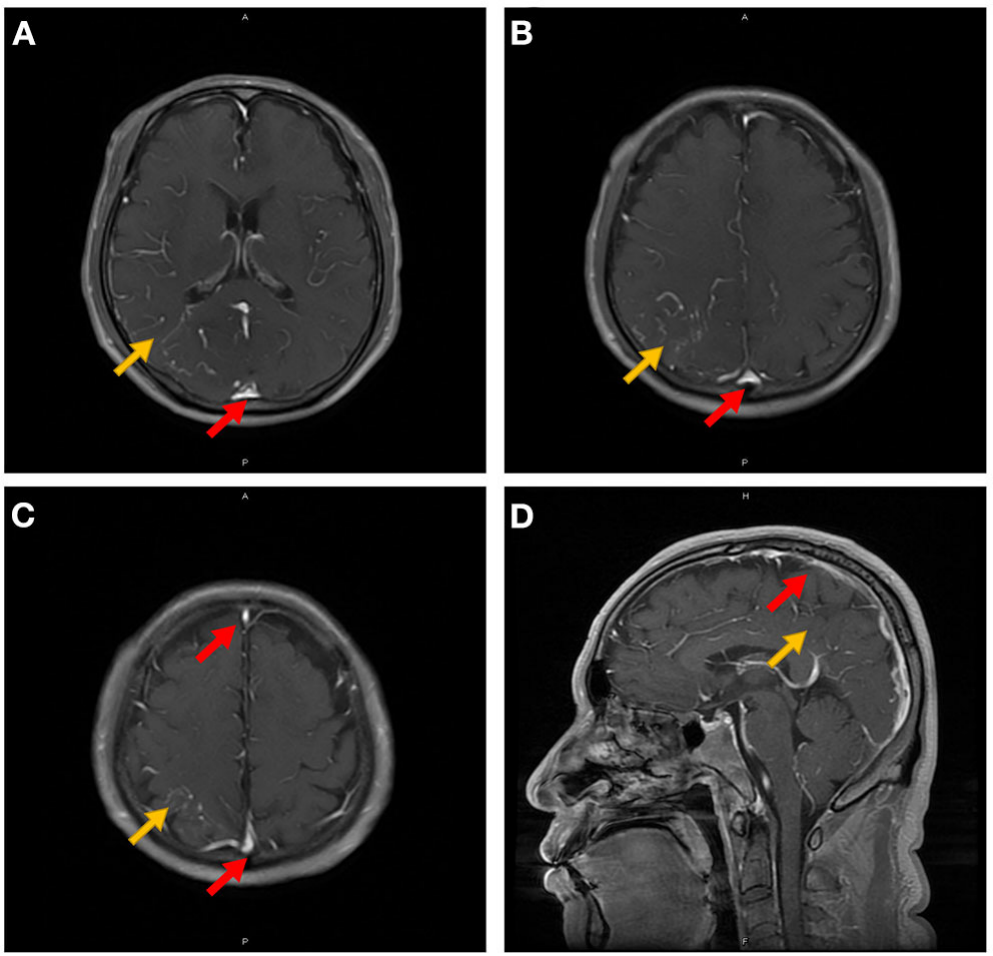
Contrast-enhanced MRI of the brain in Case 5. The orange arrow indicates leptomeningeal or medullary vascular enhancement in the right parietal-occipital lobe, and the red arrow indicates the simultaneous venous sinus enhancement **(A–D)**.

All patients underwent CT and MRI scans upon admission (Table 2). No direct evidence of CCVT was identified through these imaging modalities, despite the presence of ischemic or hemorrhagic brain lesions. However, MRI was able to identify associated pathologies in these patients, including cerebral edema (34.8%), intracerebral hemorrhages (30.4%), cortical infarctions (4.3%), SAH (13.0%) (Figure 1), and arteriovenous fistulas (AVF, 4.3%) (Figure 2).

**Table 2:** Neuroimaging patterns of the patients with CCVT on admission and follow-up.

**Case number**	**Neuroimaging on admission**	**Location of vascular and parenchymal changes based on neuroimaging studies**	**Follow-up**
1	**CT:** Left temporal and bilateral frontal hypodensity. **MRI (T1)**: Left temporal and bilateral frontal mixed signal intensity, strand-like high signal intensity near SSS, right frontal superficial cortical veins and left temporal lobe.	**Vessel:** Anterior aspect of SSS and right frontal superficial cortical veins.	**After 1-week (HR-MRBTI):** **Symptoms:** No change (PGIC = 4). **Vessel:** No change.
	**CE-MRV:** Focal narrowing LTS, LSigS, LIJV and anterior aspect of SSS. **HR-MRBTI:** Strand-like high signal intensity in SSS, LSigS, distal part of LTS, right frontal superficial cortical veins and left labbe vein.	**Brain:** HI in bilateral frontal lobe with large mass effect; brain edema surround the lesion.	**Brain:** Partial absorption of HI and post-infarction malacia within bilateral frontal lobes. **After 3-month (CE-MRV):** **Symptoms:** Partial relief (PGIC = 3). **Vessel:** Partial filling defect in bilateral parietal cortical veins, varicose left labbe vein, focal narrowing LTS, LSigS, LIJV and anterior aspect of SSS. **After 7-month (MRI+PWI):** **Symptoms:** Partial relief (PGIC = 3). **Brain:** Post-infarction malacia and low blood flow in bilateral frontal lobes.
2	**CT:** Right fronto-parietal mixed density. **MRI (T2):** Right fronto-parietal mixed density.	**Vessel:** Right fronto-parietal cortical vein.	**After 2-week (HR-MRBTI):** **Symptoms:** Partial relief (PGIC = 3).
	**MRA:** No focal narrowing in intracranial artery. **CE-MRV:** Filling defect in SSS and right fronto-parietal veins. **HR-MRBTI:** Strand-like high signal intensity in right fronto-parietal lobe and SSS.	**Brain:** HI in right fronto-parietal lobe and periventricle with large mass effect; brain edema surround the lesion.	**Vessel:** Partial recanalization. **Brain:** Post-infarction. malacia within the fronto-parietal lobe. **After 3-month (HR-MRBTI+CE-MRV+MRI):** **Symptoms:** Partial relief (PGIC = 2). **Vessel:** Partial recanalization. **Brain:** Smaller size of post-infarction malacia within the fronto-parietal lobe.
3	**CTV:** Focal narrowing LSigS. **MRI (T1):** Bilateral frontal cortex iso/hypo signal intensity. **CE-MRV:** Focal narrowing LIJV, LTS, LSigS and asymmetrical cortical veins distribution.	**Vessel:** Right frontal and left parietal cortical vein, posterior part of LTS, LSigS, and proximal part of LIJV.	**After 6-month (HR-MRBTI):** **Symptoms:** Partial relief despite acute onset of headache due to increased life pressure (PGIC = 3).
	**HR-MRBTI:** Strand-like high signal intensity in right frontal and left parietal, and right frontal low signal intensity.	**Brain:** HI in right frontal lobe.	**Vessel:** No change. **Brain:** Absorption of HI within the right frontal lobe.
4	**CTV:** No focal narrowing in intracranial veins. **MRI (T1):** Strand-like high intensity signal in bilateral frontal lobe sulcus. **CE-MRV:** Focal narrowing in anterior part of SSS.	**Vessel:** Bilateral fronto-parietal superficial vein near SSS and SSS.	**After 1-week (MRI):** **Symptoms:** Partial relief (PGIC = 3). **Vessel:** Not known.
	**HR-MRBTI:** Strand-like high intense signal in bilateral fronto-parietald lobe.	**Brain:** Cortical SAH, edema in right frontal lobe, micro-HI in left frontal lobe.	**Brain:** Smaller size of cortical SAH, edema in right frontal lobe, micro-HI in left frontal lobe. **After 4-month (CE-MRV+** **HR-MRBTI):** **Symptoms:** Complete relief (PGIC = 2) **Vessel:** Complete recanalization **Brain:** Partial absorption of SAH in left frontal lobe.
5	**CTA:** Fenestration in the A1 Segment of right anterior cerebral artery A1 segment. **MRI (T2, FLAIR, DWI):** Right parietal cortical and subcortical high signal intensity, Bilateral frontal subcortical and periventricular infarction. **CE-MRV:** Focal narrowing in RTS and abnormal collateral cortical veins formation in right side. **DSA:** Dural arteriovenous fistula in right parieto-occipital lobe.	**Vessel:** Right parietal cortical vein. **Brain:** AVF in right parieto-occipital lobe.	Non
6	**MRI (T2):** Spot-like abnormal signal surrounding lateral ventricles **CTV:** No focal narrowing in intracranial veins. **CE-MRV:** Right vertebral veins varices and abnormal bilateral collateral cortical veins formation.	**Vessel:** Bilateral SigS and right frontal cortical veins. **Brain:** Infarction surrounding lateral ventricles.	Non
	**HR-MRBTI:** Strand-like high intense signal in right frontal cortical veins and bilateral SigS **DSA:** Focal narrowing in bilateral SigS (severe).		
7	**CT:** Right fronto-temperal mixed signal density, left parieto-occipital hemorrhagic infarction.	**Vessel:** Right fronto-parietal cortical veins	**After 20-day (CTP):** **Symptoms:** Partial relief (PGIC = 3).
	**MRI (T1):** Low signal intensity in right fronto-temperal sulcus, left parieto-occipital hemorrhagic infaction. **HR-MRBTI:** Strand-like high intense signal in right fronto-parietal cortical veins.	**Brain:** HI in right fronto-temperal lobe and left parieto-occipital lobe with surrounding edema.	**Vessel:** Complete recanalization. **Brain:** Absorption of edema in left parieto-occipital lobe.
8	**CT:** Edema in right frontal cortex. **MRI (DWI):** High signal intensity in right fronto-parietal cortex. **CE-MRV:** Focal narrowing in LTS.	**Vessel:** Right fronto-parietal superficial cortical vein.	**After 4-month (CT+MRI+CTA+** **HR-MRBTI):** **Symptoms:** Complete relief (PGIC = 3).
	**HR-MRBTI:** Strand-like high intense signal in right fronto-parietal superficial cortical vein.	**Brain:** Venous infarction in right fronto-parietal lobe with surrounding edema.	**Vessel:** Focal enlargement in M2 segment of right middle cerebral artery. **Brain:** no change. **After 7-month (MRI+CE-MRV+** **HR-MRBTI):** **Symptoms:** Acute onset of right side hemiparesis (PGIC = 6). **Vessel:** No change of thrombosis in right fronto-parietal superficial cortical vein; and new onset of thrombosis in left parietal cortical veins. **Brain:** Partial absorption of HI within right fronto-parietal lobe, suspected new-onset SAH in left fronto-parietal lobe.
9	**CT:** Right frontal low signal density. **MRI (T1):** Strand-like high signal intensity in right fronto-parietal subcortex. **CE-MRV:** Focal narrowing SSS and LSigS.	**Vessel:** Bilateral fronto-parietal superficial cortical vein, posterior part of SSS and bilateral TS.	**After 9-month (MRI+CE-MRV+** **HR-MRBTI):** **Symptoms:** Acute onset of worse symptoms (PGIC = 6).
	**HR-MRBTI:** Strand-like high signal intensity in bilateral fronto-parietal subcortex, focal narrowing in posterior part of SSS and right TS. **DSA:** Focal narrowing in SSS and bilateral TS.	**Brain:** HI in right fronto-parietal and left frontal lobe.	**Vessel:** Slight partial resolution. **Brain:** Absorption of HI within right fronto-parietal and left frontal lobe. **After 21-month (CE-MRV):** **Symptoms:** Partial relief (PGIC = 3). **Vessel:** Focal narrowing in anterior part of SSS and bilateral collateral cortical veins formation.
10	**CT, MRI:** No abnormal finding. **CE-MRV:** Focal narrowing SSS. **HR-MRBTI:** Strand-like high signal intensity in right fronto-parietal cortical vein.	**Vessel:** SSS and right fronto-parietal cortical vein.**Brain:** No parenchyma lesion.	Non
11	**CT, MRI:** No abnormal finding. **CE-MRV:** Bilateral varicose cortical veins, focal narrowing RTS and RSigS **CTV:** Focal narrowing RTS and RSigS. **HR-MRBTI:** Strand-like high signal intensity in anterior part of right frontal cortical vein and right parietal cortical vein. **DSA:** Focal narrowing RTS and RSigS due to brain parenchyma compression.	**Vessel:** RTS, RSigS and right frontal and parietal superficial cortical veins. **Brain:** No parenchyma lesion.	**After 4.5 months (HR-MRBTI):** **Symptoms:** Partial relief of headache and blurry vision (PGIC = 2). **Vessel:** Partial resolution.
12	**CT:** SAH in right fronto-parietal lobe. **MRI:** Partial right fronto-parietal lobe edema. **CE-MRV:** Focal narrowing in RTS and RSigS, and abnormal collateral cortical veins formation. **HR-MRBTI:** High signal intensity in RTS and RSigS. **DSA:** No abnormal findings.	**Vessel:** RTS, RSigS and right fronto-parietal cortical vein. **Brain:** right fronto-parietal lobe edema.	Non
13	**CT, MRI:** No positive finding. **CE-MRV:** Congested right parietal cortical veins and focal narrowing in bilateral TS.	**Vessel:** Bil TS and right frontal cortical vein. **Brain:** No parenchyma lesion.	Non
	**CTV:** Enlarged arachnoid granulations in bilateral TS. **HR-MRBTI:** Strand-like high signal intensity in anterior part of right frontal cortical vein.		
14	**CT, MRI:** No positive finding. **CE-MRV:** Complete disappearance of RTS, RSigS and collateral cortical veins formation in right side.	**Vessel:** RSigS and right frontal and parietal cortical veins.	**After 1 year (CE-MRV):** **Symptoms:** No change (PGIC = 4). **Vessel:** partial narrowing in RTS, RSigS.
	**CTV:** left dominant cerebral venous system. **HR-MRBTI:** High signal intensity in right frontal cortical vein and right parietal cortical vein and high signal intensity surrounding enlarged arachnoid granulations in RSigS. **DSA:** Focal narrowing in RSigS.	**Brain:** No parenchyma lesion.	**After 2 years (CE-MRV):** **Symptoms:** No change (PGIC = 4). **Vessel:** Partial narrowing in RTS, RSigS and collateral cortical veins formation. **After 3 years (CE-MRV):** **Symptoms:** Acute onset of worse symptoms (PGIC = 6). **Vessel:** Partial narrowing in RTS, RSigS and much more collateral cortical veins formation.
15	**CT, MRI:** No positive finding. **CE-MRV:** Partial narrowing in LTS, LSigS.	**Vessel:** LTS, LSigS. **Brain:** No parenchyma lesion.	**After 2-month (HR-MRBTI):** **Symptoms:** Complete relief of blurry vision (PGIC = 3). **Vessel:** Partial narrowing in LTS, LSigS and left parietal veins. **After 9 months (HR-MRBTI):** **Symptoms:** Focal headache in the left side (PGIC = 5). **Vessel:** Complete recanalization in LTSS, LSigSS and no change of thrombosis in left parietal veins. **After 15 months (CE-MRV):** **Symptoms:** No change of focal headache in the left side (PGIC = 4). **Vessel:** Partial narrowing in LTS, LSigS and congested left labbe vein.
16	**CT:** No positive finding. **MRI (Flair):** White matter hyperdensity surrounding ventricle. **CE-MRV:** Focal narrowing in bilateral TS, SigS, J3 segment of RIJV, bilateral congested cortical veins, asymmetrical cortical vein distribution. **HR-MRBTI:** High signal intensity surrounding enlarged arachnoid granulations in anterior part of SSS and left frontal cortical vein. Enlarged arachnoid granulations in bilateral TS and SigS. **DSA:** No positive finding.	**Vessel:** SSS, Bil TSS and SigS, J3 segment of RIJV and left frontal cortical vein **Brain:** White matter hyperdensity	Non
17	**CT:** No positive finding. **MRI:** No positive finding. **CTV:** Focal narrowing in SSS, RTS, and J2 segment of RIJV (carotid artery compression).	**Vessel:** SSS, RTS, RSigS, J2 segment of RIJV right parietal cortical veins, and left frontal cortical veins	Non
	**CE-MRV:** Focal narrowing in SSS, RTS, RSigS, J2 segment of RIJV, and asymmetric distribution of cortical veins in parietal lobes. **HR-MRBTI:** Strand-like high signal intensity in SSS, RTS, RSigS, right parietal cortical veins, and left frontal cortical veins. **DSA:** Focal narrowing in RTS. (endovascular thrombectomy).	**Brain:** No parenchyma lesion.	
18	**CT:** High signal density of SSS, RTS, and SS. **MRI:** High signal intensity in RTS and RSigS. **HR-MRBTI:** Strand-like high signal intensity in SSS, SS, RTS, RSigS, right temporal cortical vein and J3 segment of RIJVS.	**Vessel:** SSS, SS, RTS, RSigS, right fronto-parietal cortical vein and J3 segment of RIJVS.	**After 20 days (HR-MRBTI):** **Symptoms:** Complete relief symptoms (PGIC = 1). **Vessel:** Partial recanalization in SSS, SS, RTS, RSigS, right temporal cortical vein.
	**DSA:** Focal narrowing in SSS and RTS. (endovascular thrombectomy).	**Brain:** Edema in bilateral side of cerebral parenchyma	**Brain:** Decrease edema size.
19	**CT:** No positive finding. **MRI:** Cortical edema in left frontal, temporal, parietal lobes.	**Vessel:** Left frontal cortical veins.	**After 20 days (HR-MRBTI):** **Symptoms:** Complete relief symptoms (PGIC = 2).
	**CTV:** Focal narrowing in SSS, LSigS and J3 segment of LIJV (bone compression). **CE-MRV:** Focal narrowing in anterior part of SSS, LTS and LIJV.		
	**HR-MRBTI:** Strand-like high signal intensity in left frontal cortical veins	**Brain:** Subcortical edema in left frontal, temporal, parietal lobes.	**Vessel:** Partial recanalization of left frontal cortical veins. **Brain:** Decrease edema size.
20	**CT:** Slight high signal density in SSS and RTS **MRI:** No positive finding. **CE-MRV:** No positive finding.	**Vessel:** SSS, RTS and right frontal cortical veins	**After 20 days (HR-MRBTI):** **Symptoms:** Complete relief symptoms (PGIC = 2).
	**HR-MRBTI:** Strand-like high signal intensity in SSS, RTS and right frontal cortical veins.	**Brain:** No parenchyma lesion.	**Vessel:** Partial recanalization in SSS, RTS and right frontal cortical veins. **Brain:** No parenchyma lesion.
21	**CT:** High signal density in RTS. **MRI:** High signal intensity in SSS. **CTV:** Focal narrowing in middle part of SSS, RSigS, and J3 segment of RIJV (bone compression).	**Vessel:** Middle part of SSS, RSigS, bilateral fronto-parietal cortical veins, and J3 segment of RIJV.	Non
	**CE-MRV:** Focal narrowing in middle part of SSS, RSigS, and J3 segment of RIJV. **HR-MRBTI:** Strand-like high signal intensity in middle part of SSS, RSigS, bilateral fronto-parietal cortical veins, and J3 segment of RIJV.	**Brain:** No parenchyma lesion.	
22	**CT:** High signal density in right fronto-parietal lobe **MRI:** High signal intensity in right fronto-parietal lobe.	**Vessel:** Bil fronto-parietal cortical veins.	Non
	**CE-MRV:** Focal narrowing in the anterior part of SSS and asymmetric distribution of fronto-parietal cortical veins. **HR-MRBTI:** Strand-like high signal intensity in multiple frontal cortical veins **DSA:** Focal narrowing in the anterior part of SSS and multiple frontal cortical veins (endovascular thrombectomy).	**Brain:** HI in right fronto-parietal lobe.	
23	**CT:** No positive finding. **MRI:** No positive finding.	**Vessel:** LSigS and left frontal cortical vein.	Non
	**CE-MRV:** Focal narrowing in LSigS. **HR-MRBTI:** Strand-like high signal intensity in left frontal cortical vein and high signal intensity surrounding enlarged arachnoid granulations in LSigS.	**Brain:** No parenchyma lesion.	

*L, left; R, right; Bil, bilateral; T, thrombosis; S, stenosis; HI, hemorrhagic infarction; SigS, sigmoid sinus, SSS, superior sagittal sinus; TS, transverse sinus; SS, straight sinus; IJV, internal jugular vein; AVF, arteriovenous fistula; CT, computer tomography; MRI, magnetic resonance imaging; CE-MRV, Contrast-enhanced magnetic resonance venography; HR-MRBTI, High resolution magnetic resonance black-blood thrombus imaging; DSA, Digital subtraction angiography; PGIC, Patient Global Impression of Change*.

Twenty-one out of the 23 (91.3%) patients underwent CE-MRV, with no direct evidence of CCVT identified. Twelve cases (52.2%) were suspicious for CCVT owing to indirect findings (i.e., the previously-mentioned pathologies on MRI and CVST on CE-MRV). The other nine patients had negative findings on CE-MRV. Despite this, all patients had direct evidence of cortical vein thrombi on HR-MRBTI. Using CE-MRV alone, the suspected CCVT diagnostic rate in this cohort was 57.1% (12/21).

Although, DSA is the gold standard for confirmation of CVST diagnoses, it might not be the best modality for the diagnosis of CCVT. Ten patients in this cohort underwent DSA. Of these cases, one was diagnosed as CVST-mediated AVF associated with CCVT, and another case was found to have thrombosis in multiple cortical veins. The CCVT diagnostic rate by DSA in this cohort was thus only 20.0% (2/10). The remaining eight patients had negative DSA findings, and the diagnosis of CCVT was only confirmed by direct evidence of cortical vein thrombus using HR-MRBTI. This novel scanning technique identified CCVT in all patients involved in this study (100%). As an additional note, nearly all of the patients in this cohort (87.0%, 20/23) had CCVT with associated CVST-related stenosis, while only three cases demonstrated isolated CCVT without any evidence of CVST.

### Treatment and Outcomes

All patients received standard anticoagulation therapy with low molecular weight heparin (LMWH), and were then bridged to warfarin with maintenance of the international normalized ratio (INR) of 2–3 for a minimum of 6 months. Patients with seizures were also treated with anti-seizure agents. Thirteen patients had follow-up HR-MRBTI at least 2 weeks after the initial presentation, and all of them acquired complete or partial recanalization of CCVT. All patients had good functional outcomes (mRS = 0–1) after 6-month of standard anticoagulation treatment. No patients experienced bleeding events throughout the entire treatment process. No significant adverse events were observed in patients with either acute or chronic CCVT after HR-MRBTI scanning.

Four patients with CCVT involving multiple cortical veins underwent intravenous batroxobin as an affiliative therapy because anticoagulation (*n* = 3) or thrombectomy (*n* = 1) treatments were not effective. They reported symptom relief after 5IU of batroxobin use every other day. Thrombophilia tests and HR-MRBTI were carried out during follow-up visits. We also observed that fibrinogen decreased, and that degenerative products of fibrin (such as D-dimers) increased in the first 24 h following batroxobin treatment. Further, follow-up HR-MRBTI post-1-week batroxobin use demonstrated partial or complete CCVT recanalization in all four patients (**Figures 5A,B**). We also conducted a subgroup analysis of patients who had or had not been treated with batroxobin and had follow-up data (*n* = 13) (Table 3). The use of batroxobin (*n* = 4) was correlated with improved symptoms, decreased time for symptom relief, accelerated recanalization, and reduced recurrence of CCVT. However, a significant difference was only found in PGIC scores between the batroxobin group and the non-batroxobin group (*p* = 0.030) due to the small sample size.

**Table 3:** Subgroup analysis of prognosis in patients with/without batroxobin use.

**Group**	**Case number**	**Prognosis evaluation**			
		PGIC score^*^	**Time to achieve symptom relief (days)^#^**	**Recanalization of CCVT in the neuroimaging (Yes/No)**	**Recurrence of CCVT in the neuroimaging (Yes/No)**
**With batroxobin use (*****n*** **=** **4)**		1.75 ± 0.50^†^	37.50 ± 35.00^‡^	(4/0)^§^	(0/4)^∧^
Thrombectomy + Anticoagulation (Rivaroxaban) + Batroxobin	18	1	20	Yes	No
Anticoagulation (Rivaroxaban) + Batroxobin	11	2	90	Yes	No
Anticoagulation (Rivaroxaban) + Batroxobin	19	2	20	Yes	No
Anticoagulation (Dabigatran/Rivaroxaban) + Batroxobin	20	2	20	Yes	No
**Without batroxobin use (*****n*** **=** **9)**		4.00 ± 1.73	100.00 ± 57.88	(3/6)	(4/5)
Thrombectomy + Anticoagulation (Warfarin)	9	6	NA	No	Yes
Stenting + Anticoagulation (Dabigatran)	15	5	NA	No	No
Anticoagulation (Dabigatran)	1	3	90	No	No
Anticoagulation (Dabigatran)	2	2	90	Yes	No
Anticoagulation (Dabigatran)	4	2	120	Yes	No
Anticoagulation (Rivaroxaban)	14	6	NA	No	Yes
Anticoagulation (Warfarin)	3	3	180	No	Yes
Anticoagulation (Warfarin)	8	6	NA	No	Yes
Anticoagulation (Heparin)	7	3	20	Yes	No

*PGIC, Patient Global Impression of Change; NA, Not Applicable*.

* *PGIC score was evaluated at outpatient follow-up. The median PGIC score was selected when patients with multiple times follow-ups. PGIS score of subgroup was presented as mean ± SD*.

# *Time to achieve symptom relief was defined as the period from first day of clinical intervention of CCVT to the first time of self-reported symptom relief at outpatient follow-up*.

† *p = 0.030*.

‡ *p = 0.101*.

§ *p = 0.070*.

∧ *p = 0.228*.

## Discussion

Diagnosing CCVT (as a subtype of CVT) is challenging because of its non-specific symptoms and the relatively small size of cortical veins which make precise imaging difficult. Coutinho et al. found that the most successful diagnostic tool for CCVT was CE-MRV (which had a 73% accuracy rate), followed by conventional angiography (47%) (6). However, both CE-MRV and DSA diagnose CCVT indirectly, using metrics such as poor blood flow or contrast interruption, and cannot directly demonstrate cortical vein thrombosis (2).

Here, we used HR-MRBTI to directly detect CCVT. This new MRI sequence was found to be highly accurate for identifying thrombus in cortical veins, and was particularly useful for confirming CCVT in patients with false-negative CE-MRV or DSA. To our knowledge, this is the first study to evaluate the diagnostic value of HR-MRBTI for CCVT.

### Diagnostic Value of HR-MRBTI for Detecting CCVT

MRBTI, also known as MR direct thrombus imaging (MR-DTI) (16), was first developed in a swine model of carotid thrombosis by Corti et al. (17). MRBTI uses sequences of black-blood T1-weighted and T2-weighted MRIs to assess thrombus age based on differing oxygenation states of erythrocytic hemoglobin in the thrombus and changes in the intracellular/matrix content of proteins and RBC hydration over time. These double inversion recovery techniques can effectively negate the blood flow signal and delineate structures such as the vascular walls and thrombi from the lumen.

As reviewed recently by van Dam et al. (18), MRBTI has recently been used to visualize the lumen and outer wall boundaries of large arteries and venous sinuses (such as the coronary arteries, cerebral arteries, and CVS) (13, 19–21). This imaging method has great value for diagnosing complex cases of CVT.

In this study, we improved the MRBTI sequence by combining it with high-resolution MRI. The slice thickness of HR-MRBTI (0.6–1.0 mm) is much smaller than that of normal MR-DTI (2 mm), with a similar scanning time (6–8 vs. 5 min 59 s) (22). However, while HR-MRBTI was previously used to identify CVST, no prior study had examined its utility for identifying CCVT. We performed HR-MRBTI scans in patients with CCVT, identifying cortical vein thrombus in various pathological stages with a sensitivity rate of 100%. The MRBTI procedure also appeared to be safe in patients with both acute and chronic CCVT, as there were no significant adverse events after scanning. Compared to HR-MRBTI, conventional CE-MRV screening only detected CCVT in 66.7% of patients with positive indirect symptoms (i.e., asymmetrically dilated cortical veins and/or abnormal collateral cortical vein formation). When using CE-MRV, it is difficult to distinguish anatomic variations from the abnormal compensatory cortical veins in CCVT (23–26). Furthermore, DSA detection of CCVT is also based on indirect signs—including focal narrowing and slow blood flow in the CVS, and had a diagnostic rate of only 14.3% in this cohort. DSA is also limited in that it is an invasive test, has a high cost, involves radiation exposure and the use of iodinated contrast, and is contraindicated in patients who are pregnant or have iodine allergies or thyroid disease. HR-MRBTI is not only a qualitative method for evaluating pathological stages of thrombus development, but is also a quantitative way to demonstrate thrombus load (13, 27).

### Predictive Value of HR-MRBTI Combined With D-Dimers for Identifying CCVT Pathophysiological Stage

HR-MRBTI involves two scanning sequences: non-enhanced and contrast-enhanced. Imaging evaluation of CCVT using HR-MRBTI is similar to how CVST is evaluated. A newly formed thrombus (acute stage) displays high signal intensity in the non-enhanced sequence and is dark gray in the enhanced imaging sequence. Thrombi at this stage tend to have homogeneous signal intensities. Thrombi in the subacute stage are isointense on both non-enhanced and enhanced sequences, have equal signal intensity to brain parenchyma. Finally, thrombi in the chronic stage are isointense in non-enhanced sequences, and display vivid contrast enhancement.

Thrombus-mediated vessel wall inflammation and edema are widespread and may not be limited to the focal segments of visualized thrombi. Rather, it is likely that this phenomenon also affects other segments of the intracranial vasculature. Vessel wall inflammation and edema may also display high signal intensity in non-enhanced HR-MRBTI sequences, and could be easily confused with new thrombi. When this occurs, abnormally elevated plasma D-dimers are helpful for supporting the diagnosis of new thrombus formation, with the exclusion of non-thrombotic vasculitis (12). In this study, 80% of patients with CCVT in the acute stage had increased plasma D-dimer levels, while patients with CCVT in the subacute or chronic stage showed no D-dimer elevation. Thus, clinical presentations of abnormally elevated plasma D-dimers would raise the possibility of acute CCVT. HR-MRBTI, however, was more accurate for diagnosing CCVT. The combination of HR-MRBTI indications and elevated plasma D-dimers may most accurately confirm the presence of an acute CCVT.

### Probable Mechanisms of CCVT Formation

**Figure 3:**
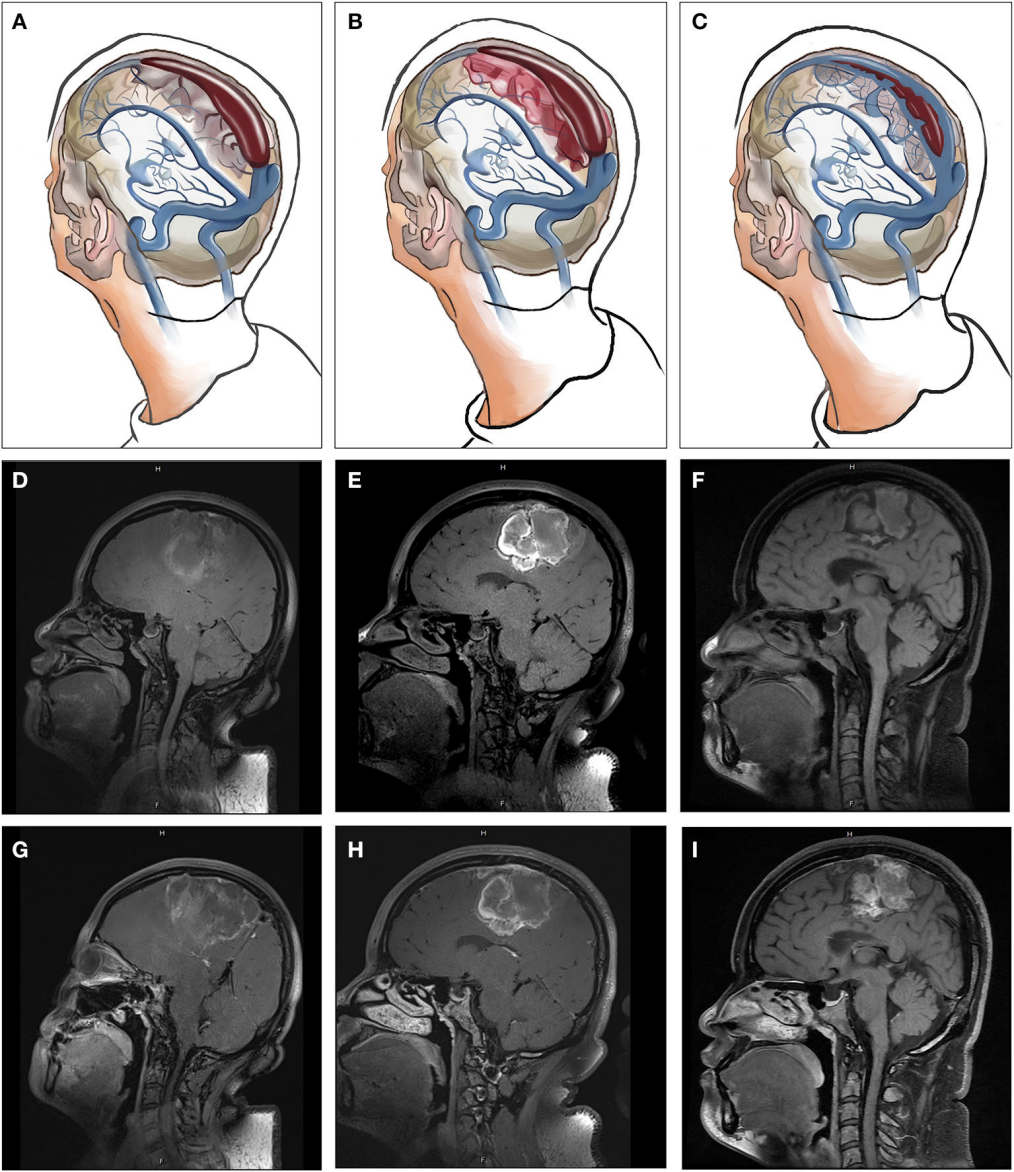
The relationships between CCVT and CVSS **(A–C)**, Non-enhanced **(D–F)**, and contrast-enhanced **(G–I)** HR-MRBTI of the brain in Case 3 (on admission). Initially, venous drainage insufficiency may induce enlargement of arachnoid granulations within the sinuses, resulting in hemodynamic changes in the sinuses and resultant endothelial damage and dysfunction, which can then lead to CVSS. Thrombosis may develop in cortical veins near CVSS. Finally, under standard therapy with anticoagulation, CCVT and CVSS may experience partial or full recanalization.

Previous case reports and case series have described CCVT as isolated cortical venous thrombosis (2). However, we found that the majority of CCVT cases were comorbid with CVST (15/16). Only one CCVT case occurred in an isolated cortical venous lumen. This frequent comorbidity suggests that CCVT may be formed because of venous sinus mural thrombus extension to the cortical venous system. Alternatively, CCVT may develop as the sinus stenosis impedes cortical venous outflow, with this stagnation resulting in cortical venous thrombosis *in situ*. It is also possible that the condition may begin in the cortical veins and then spread to larger superficial veins, before eventually involving the dural sinus walls. The interactive relationship between CVSS and CCVT could also result from a shared pathophysiological mechanism, such as hypercoagulation. Thus, further prospective studies are required to evaluate possible mechanisms, risk factors, and outcomes for patients with isolated CCVT and patients with comorbid CCVT and CVST (Figure 3).

### Clinical Manifestations of CCVT and Outcomes After Combined Anticoagulation and Batroxobin Therapy

**Figure 4:**
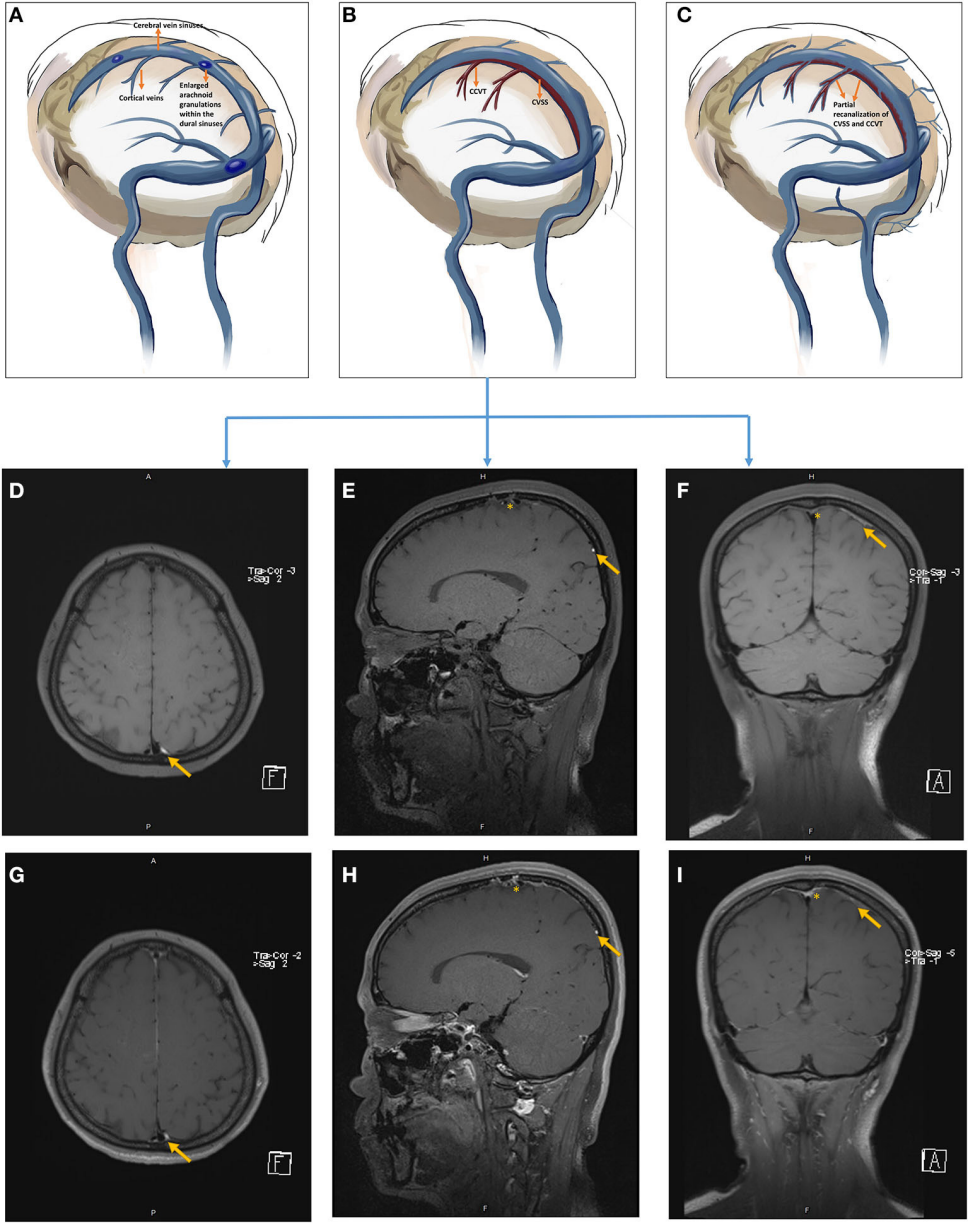
The pathophysiology of hemorrhagic transformation associated with CCVT **(A–C)** and follow-up of non-enhanced **(D–F)** and contrast-enhanced **(G–I)** HR-MRBTI of the brain in Case 2 (on admission, **D,G**; 2-week follow-up, **E,H**; 6-week follow-up, **F,I**). The orange asterisk represents CVSS, and the orange arrow indicates CCVT. Initially, CCVT was accompanied by surrounding edema **(A,D,G)**; but hemorrhagic transformation would likely arise if standard anticoagulation and hydration therapy were not administered appropriately **(B,E,H)**. Parenchymal edema and the hematoma were gradually absorbed, and the lesion underwent neural recovery **(C,F,I)**.

CCVT has various clinical manifestations and lacks specific features in routine imaging. It is easily misdiagnosed, which often results in treatment delay. Headache and seizures are the most common symptoms reported in previous case reports (1, 2). These findings were replicated in our study, with seizures and headaches seen in 43.8 and 62.5% of patients, respectively. Associated brain tissue lesions were found in 68.8% of patients. Common initial neurologic manifestations of CCVT were ischemic stroke, hemorrhagic transformation, cerebral edema, and SAH (Figure 4). These manifestations are easily diagnosed, and but the underlying CCVT is often missed (8, 28, 29).

Atypical features on CT or MRI scans, such as cerebral infarctions which cross typical arterial territories, and hemorrhagic infarctions or lobar cerebral hemorrhages with unclear etiologies, may indicate underlying CVT (30). CE-MRV has excellent diagnostic performance and is accurate for confirming CVST and deep cerebral vein thrombosis (31, 32). However, its sensitivity for detecting CCVT is poor when compared to HR-MRBTI.

Severe intracranial hypertension (defined as lumber open puncture pressure over 330 mmH_2_O) is infrequently seen (<20%) with CCVT, much lower than in CVST (6, 33). For patients with elevated ICP, optical coherence tomography (OCT), ophthalmoscopy, and ophthalmologic interventions (such as optic nerve sheath decompression) are necessary to avoid permanent visual damage (34).

The underlying etiology of CCVT remains unclear. Venous thrombosis should be highly suspected in young female patients with associated brain lesions and who have medical histories which include hyperlipidemia, diabetes mellitus, oral contraceptive use, obesity, autoimmune diseases, blood clotting disorders, and/or cancer chemo/radiotherapy. Autoimmune diseases such as systemic lupus erythematosus (SLE), antiphospholipid syndrome, and vasculitis are frequently seen alongside CCVT (35–38). Unsurprisingly, we found that CCVT was strongly associated with hypercoagulable states (hyperfibrinogenemia, AT-III deficiency, protein S deficiency, and protein C deficiency). Thus, a workup for autoimmune diseases and thrombophilia in these patients is highly recommended. Abnormal thyroid function testing was also observed in this patient cohort, indicating hyperthyroidism as a potential underlying factor that influences the development of CCVT. This is consistent with previous reports which found that hyperthyroidism may contribute to CVST (39–42).

**Figure 5:**
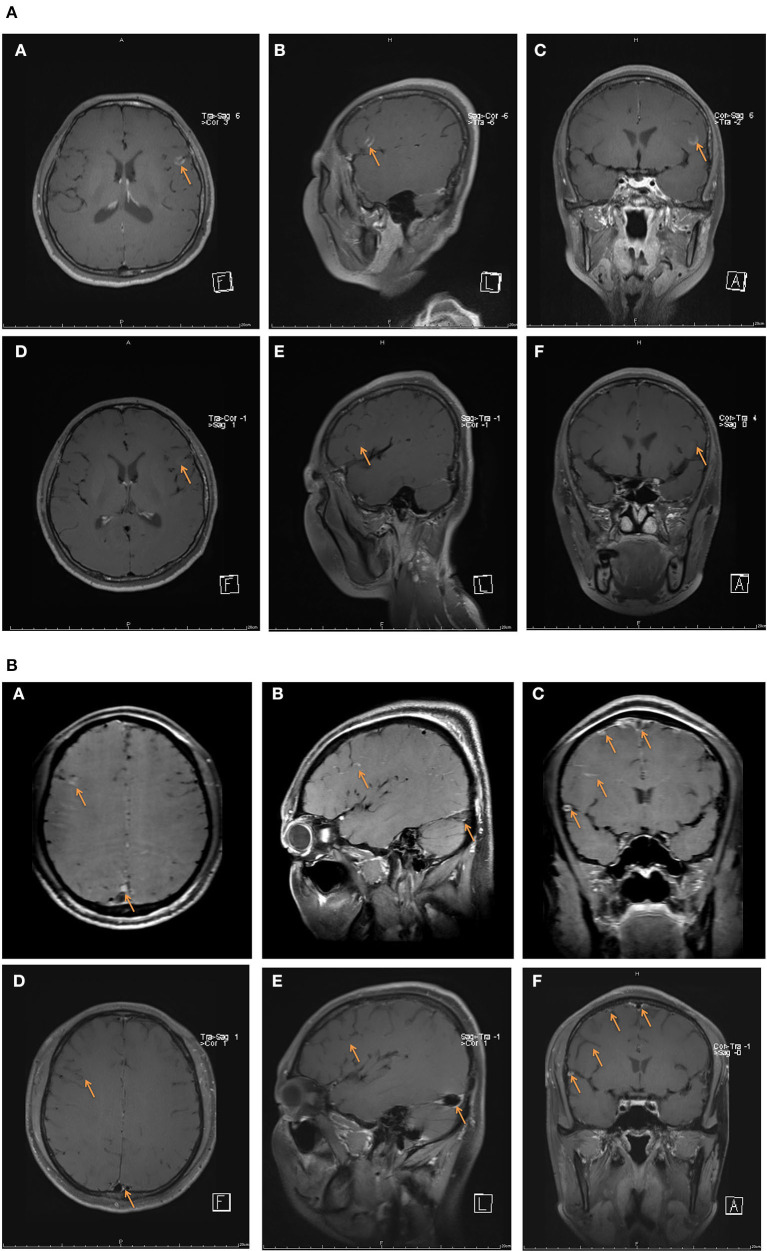
Contrast-enhanced HR-MRBTI of the brain during the pre-batroxobin period (upon admission) and post-batroxobin period (2-week follow-up). **(A)** Case 19: a thirty-seven-year-old female with thrombosis in multiple sites of the left frontal lobe was given batroxobin as well as anticoagulation therapy. **(A–C)** pre-batroxobin period (upon admission), **(D–F)** post-batroxobin period (2-week follow-up). **(B)** Case 18: a thirty-one-year-old male with CVST in multiple sinuses and thrombosis in multiple sites of right frontotemporal lobes was administrated batroxobin after a two-time thrombectomy of CVST. **(A–C)** pre-batroxobin period (upon admission), **(D–F)** post-batroxobin period (2-week follow-up).

Long-term standardized anticoagulation is widely accepted as the first-line strategy for CVST. However, our previous study indicated that multitherapy of batroxobin and anticoagulation treatments could reduce the restenosis rate and accelerate cerebral venous recanalization compared with anticoagulation monotherapy in patients with CVST (14, 15). In the clinical setting, we found that some patients with CCVT still experienced symptoms despite long-term anticoagulation treatment. This may be because of the relatively low distribution of anticoagulants in cortical veins, which are small in size and have poor circulatory properties. Thus, we selected four CCVT patients that had cases involving multiple cortical veins to take intravenous batroxobin combined with oral anticoagulation agents. Surprisingly, all of them experienced immediate symptom relief and recanalization of CCVT at 1-week post-treatment. Further, we observed that the combined use of batroxobin and anticoagulation therapy (*n* = 4) correlated with improved symptoms, decreased time to symptom relief, increased rates of recanalization, and reduced recurrence of CCVT compared to anticoagulation monotherapy (*n* = 9). However, there was only a statistically significant difference between PGIC scores in the batroxobin group and the non-batroxobin group (*p* = 0.030) due to the small sample size and considerable variety in an anticoagulation/endovascular therapy. No bleeding events were reported in these four cases throughout the treatment process. Thus, intravenous batroxobin may be a safe and effective affiliative therapy in combination with anticoagulants (Figure 5A) or thrombectomy (Figure 5B). Future studies with a larger sample size are needed to further evaluate the efficacy and safety of batroxobin for treating CCVT.

**Figure 6:**
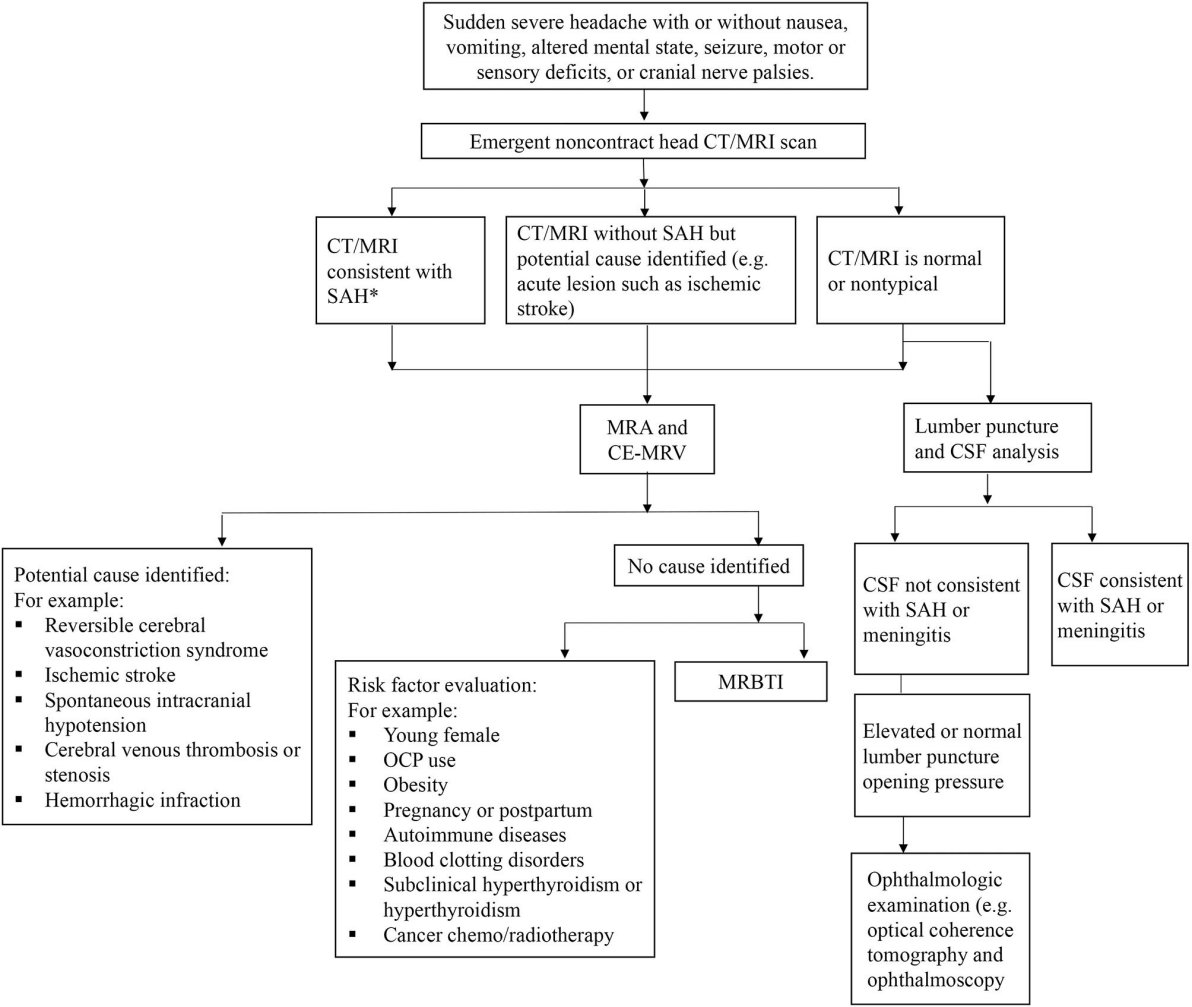
Algorithm of management for CCVT.

In this cohort, the majority of CCVT patients had good outcomes at both discharge and outpatient follow-up. HR-MRBTI and D-dimer levels were useful for evaluating the response to therapy in outpatient follow-up. We propose a diagnostic and management algorithm for suspected CCVT based on our clinical experiences (Figure 6).

## Limitations

There are several limitations to this study. First, we had a small sample size, although that is partially due to the rarity of the disease. Although we tried to enroll all eligible patients for this study, many patients with suspected CCVT were not included because of our stringent criteria (i.e., that either DSA or CE-MRV images needed to have been collected at the same time as HR-MRBTI imaging). Second, all patients were enrolled and evaluated during inpatient hospitalization, and as such, likely had relatively severe forms of the condition which required aggressive treatment. Moreover, HR-MRBTI, as a special sequence of high resolution-MRI is still not commonly utilized in most hospitals and is not generalizable to many clinical settings. However, conventional neuroimaging could help exclude many other differential diagnoses in the early steps. Patients with new-onset headaches or seizures should be flagged as potential CCVT cases. Finally, CCVT was strongly related to CVST. Further studies will be needed to more comprehensively explore the relationship between these two conditions.

## Conclusions

HR-MRBTI may be a fast and accurate imaging tool for non-invasive CCVT diagnosis. HR-MRBTI combined with examination of D-dimer levels could precisely identify the pathological stage of CCVT. Batroxobin may safely accelerate cortical venous recanalization as an affiliative therapy for anticoagulation or endovascular therapy. Future studies with a larger sample size are needed to further evaluate the safety and efficacy of batroxobin as a treatment for CCVT.

## Data Availability Statement

The raw data supporting the conclusions of this article will be made available by the authors, without undue reservation.

## Ethics Statement

The studies involving human participants were reviewed and approved by Ethics Committee of Xuanwu Hospital, Capital Medical University. The patients/participants provided their written informed consent to participate in this study.

## Author Contributions

S-yS: manuscript drafting and revision, data collection, assembly, interpretation, and picture drawing. DD and Y-cD: manuscript drafting and revision. DL, B-lJ, S-lW, and Y-bG: data collection, assembly, and interpretation. QY: data assembly and interpretation. X-mJ: study concept and design. RM: study concept, design, manuscript drafting, and revision. All authors contributed to the article and approved the submitted version.

## Conflict of Interest

The authors declare that the research was conducted in the absence of any commercial or financial relationships that could be construed as a potential conflict of interest.
