# Supplementary material for: High-Resolution Magnetic Resonance Black Blood Thrombus Imaging and Serum D-Dimer in the Confirmation of Acute Cortical Vein Thrombosis
Source: Front Neurol. 2021 Jun 21;12:680040. doi: 10.3389/fneur.2021.680040 (PMC8255931; doi:10.3389/fneur.2021.680040)
Supplement: Supplementary file 1 [file Table_1.docx]

**Supplementary Table 1** Reference interval for laboratory test results

| **Values** | Reference interval |
| --- | --- |
| **Blood sample** |  |
| WBC count | 4.0-10.0 *10^9/L |
| Neutro count | 1.8-6.4 *10^9/L |
| PLT | 100-300 *10^9/L |
| RDW | 0.0-15.0% |
| Hb | 110-150 g/L |
| Fibrinogen | 2.0-4.0 g/L |
| DD | 0.01-0.5 μg/mL |
| PS | 55.0-123.0% |
| PC | 65.0-140.0% |
| AT-Ⅲ | 80.0-120.0% |
| CRP | 1.0-8.0 mg/L |
| Hs-CRP | 0.0-3.0 mg/L |
| ESR | 0.0-20.0 mm/h |
| C3 | 0.79-1.52 g/L |
| C4 | 0.16-0.38 g/L |
| RF | 0.0-20.0 IU/mL |
| APS | 0.0-12.0RU/ml |
| T3 | 2.3-4.2 pg/mL |
| T4 | 0.89-1.76 ng/dL |
| Tg-Ab | 0.0-4.0 IU/mL |
| TPO-Ab | 0.0-9.0 IU/mL |
| TG | 0.45-2.25 mmol/L |
| Total cholesterol | 3.24-5.7 mmol/L |
| LDL | 2.08-3.12 mmol/L |
| HDL | 1.08-1.91 mmol/L |
| NSE | 0.0-17.0 ng/mL |
| UC | 155-416 μmol/L |
| Homocysteine | ＜15mmol/L |
| **CSF sample** |  |
| WBC count | ＜10 *10^6/L |
| Neutro count | ＜10 *10^6/L |
| Protein | 15.0-45.0 mg/dL |
| Glucose | 45.0-80.0 mg/dL |

WBC= white blood cell; Neutro=Neutrophil; Lym=lymphocyte; PLT=platelet; Hb= hemoglobin; RBC= red blood cell; CRP= C-reactive protein; IL-6=Interleukin-6; Hs-CRP= high-sensitivity C-reactive protein; DD= d-dimer; PS = Protein S; PC = Protein C; AT-Ⅲ=antithrombin-Ⅲ; T3=triiodothyronine; T4= Thyroxine; Tg-Ab= thyroglobulin antibodies; NSE = Neuron-specific enolase; TPO-Ab=Thyroid peroxidase antibody; ANA=antinuclear antibody; ESR= erythrocyte sedimentation rate; TG= triglycerides; LDL= Low-density lipoprotein cholesterol; HDL= High-density lipoprotein cholesterol; UC= uric acid; APS= Antiphospholipid syndrome; RF = rheumatic factor
